# Nitric-acid

**Published:** 1896-11

**Authors:** 


### HOMŒOPATHIC PHYSICIAN,

A MONTHLY JOURNAL OF

HOMŒOPATHIC MATERIA MEDICA AND CLINICAL MEDICINE.

"If our school ever gives up the strict inductive method of Hahnemann, we are lost, and deserve only to be mentioned as a caricature in the history of medicine."—CONSTANTINE HERING.

Vol. XVI. NOVEMBER, 1896. No. 11.

### EDITORIAL.

NITRIC-ACID.—The lustreless sunken eyes of Nitric-acid suggest also Sulphur. The spots on the cornea are similar to Calcarea-carbonica. The paralysis of the upper eyelids remind us of Phosphorus and Causticum. Drooping of the upper eyelid is Dr. Guernsey's key-note of Causticum. His expression is: "Her eyelids are so heavy she cannot keep them up; they seem paralyzed, Causticum."

The discharge of pus from the ears suggests Pulsatilla and Calcarea-carbonica as well as Nitric-acid. The echo in the ears of one's own speech suggests Causticum. The redness of the tip of the nose suggests Aloes, Carbo-vegetabilis, and Sulphur.

The following additional comparisons are offered by the Editor:

Nose red in young women. Borax.

Nose of copper-red color. Cannabis-indica.

Tip of the nose red and knobby. Aurum-metallicum.

Itching of the tip of the nose. Silicea.

Eruption of pimples upon the tip of the nose. Causticum.

Crawling sensation in the tip of the nose. Moschus.

The child rubs the nose with the fist or on the shoulder of the nurse. Cina.

The child wakens at night and rubs the nose so much and so long that the parents are frightened. It won't stop rubbing its nose. Lycopodium. (*Guiding Symptoms.*)

Nitric-acid has sneezing during sleep.

Nitric-acid has coryza, hoarseness, and sore throat. The nose is stopped up, and yet it runs water. Head feels like a board, and the patient feels beaten and bruised all over.

In conversation with Dr. Lippe in September, 1881, he spoke of the peculiar coryza of Nitric-acid, which had been illustrated by a case he had just cured. He said that Nitric-acid is almost certainly the remedy where we had the three symptoms together, coryza, hoarseness, and sore throat. That the running of water from the nose, which is at the same time stopped up, is characteristic of Nitric-acid.

Scurf in the nose is under Calcarea-carbonica as well as Nitric-acid. Sticta-pulmonaria has the characteristic, the secretions from the nose dry up so rapidly that they form crusts upon the entrances to the nostrils. Complete obstruction of the nose is characteristic of Nitric-acid, but also occurs under Lycopodium and Silicea.

Nitric-acid has mucus dropping through the posterior nares. This is the great characteristic of Kali-bichromicum.

The soreness of the throat in coryza, under Nitric-acid, is a stitching soreness.

Petroleum has fluent coryza and hoarseness.

Nitric-acid has bloated condition around the eyes on waking early. This is similar to Apis.

Nitric-acid has swelling of the lips. Calcarea-carbonica has swelling of the upper lip. Apis has the same symptom.

Nitric-acid is useful in painful swelling of the submaxillary glands. This is similar to Calcarea-carbonica.

Pain in hollow teeth, especially after the abuse of Mercury, indicates Nitric-acid.

Under Nitric-acid the gums are white, swollen, and bleeding. This is similar to Mercurius. Dryness of the mouth with thirst indicates Nitric-acid and Natrum-muriaticum.

Nitric-acid has ptyalism, like Mercury. Phosphorus has bloody ptyalism.

Sensitive tongue, with smarting sensation, even from mild food, indicates Nitric-acid and Natrum-muriaticum. A white, dry tongue indicates Nitric-acid and Apis.

Violent thirst in the morning in those suffering from suppuration of the lungs, indicates Nitric-acid. This, according to Dr. Lippe, is a clinical observation of Hahnemann.

Perspiration during and after eating indicates Nitric-acid and Natrum-carbonicum.

The Nitric-acid patient gets water-brash from drinking fast.

Pain in the cardiac orifice of stomach on swallowing food indicates Nitric-acid. It also indicates Phosphorus, Alumina, and Bryonia.

When the Nitric-acid takes cold it generally settles in the abdomen and causes colic. This is characteristic of Nitric-acid.

Incarcerated flatulence in the upper abdomen indicates Nitric-acid. Aloes and Lycopodium also have it, but under Lycopodium it is worse at night.

Nitric-acid has stitches and pricking in the rectum, with tenesmus after stool.

Nitric-acid is indicated in children who are troubled with incontinence of urine, and so is Causticum.

Nitric-acid is particularly useful in inflammation of the prepuce with swelling and phymosis. Mercury and Sulphur are also indicated. In the Nitric-acid patient the testicles hang down too low.

Under Nitric-acid the menses are too early, like Calcarea. During menses there is colic, and this also indicates Natrum-carbonicum.

Nitric-acid has dry tickling cough on lying down, so also has Calcarea-carbonica.

Cramp-like pain in the chest under Nitric-acid also suggests Cocculus.

Nitric-acid has soreness of chest from deep breathing and coughing. Calcarea-carbonica has soreness of the chest when touching it. This is an excellent indication for Calcarea.

